# Men Living with Diagnosed HIV Who Have Sex with Men: Progress Along the Continuum of HIV Care — United States, 2010

**Published:** 2014-09-26

**Authors:** Sonia Singh, Heather Bradley, Xiaohong Hu, Jacek Skarbinski, H. Irene Hall, Amy Lansky

**Affiliations:** 1 Division of HIV/AIDS Prevention, National Center for HIV/AIDS, Viral Hepatitis, STD, and TB Prevention, CDC

Gay, bisexual, and other men who have sex with men (MSM) represent approximately 2% of the United States population, yet are the risk group most affected by human immunodeficiency virus (HIV) (1). In 2010, among persons newly infected with HIV, 63% were MSM (2); among persons living with HIV, 52% were MSM (3). The three goals of the National HIV/AIDS Strategy are to reduce new HIV infections, to increase access to care and improve health outcomes for persons living with HIV, and to reduce HIV-related health disparities (4). In July 2013, the HIV Care Continuum Initiative was established by executive order to mobilize and accelerate federal efforts to increase HIV testing, services, and treatment along the continuum (5). To meet the 2015 targets of the National HIV/AIDS Strategy, 85% of MSM diagnosed with HIV should be linked to care, 80% should be retained in care, and the proportion with an undetectable viral load (VL) should be increased by 20%. To assess progress toward meeting these targets, CDC assessed the level at each step of the continuum of care for MSM by age and race/ethnicity. CDC analyzed data from the National HIV Surveillance System (NHSS)* and the Medical Monitoring Project (MMP)† for MSM with diagnosed HIV infection. The results indicated that 77.5% were linked to care, 50.9% were retained in care, 49.5% were prescribed antiretroviral therapy (ART), and 42.0% had achieved viral suppression. Younger MSM and black/African American MSM had lower levels of care compared with older MSM and those of all other races/ethnicities. Interventions aimed at MSM are needed that increase linkage to care, retention in care, and ART use, particularly among MSM aged <25 years and black/African American MSM.

Data from NHSS in 2010, reported to CDC through December 2012, were used to determine the numbers of MSM aged ≥13 years newly diagnosed and living with HIV and the numbers and percentages linked to care and retained in care. Nineteen jurisdictions met the criteria for the collection and reporting of CD4+ T-lymphocyte (CD4) and VL test results,§ the data used to assess linkage and retention in care. Linkage to care¶ was estimated among MSM with new HIV diagnoses during 2010 who resided in any of the 19 jurisdictions at diagnosis. Retention in care** was assessed among MSM with HIV diagnosed by December 31, 2009, who resided in any of the 19 jurisdictions at diagnosis and were alive on December 31, 2010. Data were statistically adjusted for missing HIV transmission categories (6).

Data from MMP were used to estimate ART prescription†† and viral suppression§§ among MSM aged ≥18 years using methods described previously (7). The MMP values are weighted national estimates of the numbers of MSM who received medical care during January–April 2010 and had documentation of ART prescription and viral suppression. Percentages were estimated among MSM whose HIV infection was diagnosed by December 31, 2009, and who were alive on December 31, 2010, in the United States and Puerto Rico (denominators were based on NHSS data). Data analyses were limited to 2010, the most recent year for which data were available for persons living with HIV infection.

Of the 10,093 MSM with HIV infection diagnosed during 2010 in the 19 jurisdictions, 7,826 (77.5%) were linked to care within 3 months after HIV diagnosis (Table 1). The percentage linked to care increased with age. Those aged 13–24 years had the lowest percentage of linkage to care (71.0%), and those aged 45–54 years had the highest percentage (84.1%). By race/ethnicity, black/African American MSM had the lowest percentage of linkage to care (71.6%), followed by Hispanic/Latino (80.3%) and white MSM (82.9%).

Among the 174,071 MSM aged ≥13 years living with diagnosed HIV on December 31, 2010, in 19 jurisdictions, 50.9% were retained in care (Table 2). Retention in care also increased with age. Those aged 13–24 years had the lowest percentage of retention in care (45.7%), and those aged ≥55 years had the highest percentage (53.5%). By race/ethnicity, black/African American MSM had the lowest percentage of retention in care (46.3%), followed by white (52.1%) and Hispanic/Latino MSM (54.1%).

Of 416,730 MSM aged ≥18 years living with diagnosed HIV infection on December 31, 2010, in the United States and Puerto Rico, 206,461 (49.5%) were prescribed ART (Table 3). The prevalence of ART prescription increased with age. Among those aged 18–24 years, 30.5% were prescribed ART, and among those aged ≥55 years, 67.7% were prescribed ART. By race/ethnicity, black/African American MSM had the lowest level of ART prescription (47.1%), followed by Hispanic/Latino (49.2%) and white MSM (49.6%).

Among the 416,730 MSM living with diagnosed HIV in the United States and Puerto Rico, 42.0% achieved viral suppression at their most recent test (Table 3). Levels of viral suppression increased with age. Those aged 18–24 years had the lowest level of viral suppression (25.9%), and those aged ≥55 years had the highest level (60.8%). By race/ethnicity, black/African American MSM had the lowest level of viral suppression (37.0%), followed by Hispanic/Latino (41.5%) and white MSM (43.9%).

## Discussion

A high percentage of MSM diagnosed with HIV in 2010 were linked to care (77.5%); however, only 50.9% of MSM living with diagnosed HIV infection were retained in care, 49.5% were prescribed ART, and 42.0% had achieved viral suppression. Increasing access to care and sustained treatment is critical for improving health outcomes and to reduce the potential of transmitting HIV among MSM, who are the majority of persons with HIV infection.

What is already known on this topic?

Gay, bisexual, and other men who have sex with men (MSM) represent approximately 2% of the United States population, yet represent 63% of new HIV infections.

What is added by this report?

In 2010, for MSM with diagnosed HIV infection, 77.5% were linked to care, 50.9% were retained in care, 49.5% were prescribed antiretroviral therapy, and 42.0% had achieved viral suppression. At each step of the continuum of care, younger MSM had lower levels of care compared with older MSM. Black/African American MSM had the lowest levels of care compared with MSM of all other races/ethnicities.

What are the implications for public health practice?

Interventions aimed at MSM that increase care, particularly among MSM aged <25 years and black/African American MSM are needed to achieve the goals of the National HIV/AIDS Strategy to reduce new HIV infections, to increase access to care and improve health outcomes for persons living with HIV, and to reduce HIV-related health disparities.

At each step of the continuum of care, younger MSM had lower levels of care compared with older MSM. Black/African American MSM had the lowest levels of care compared with those of all other race/ethnicities. Lack of health insurance, stigma, and discrimination might influence whether MSM access medical care, which has implications for each subsequent step along the continuum of care. Implementing effective interventions for young MSM aged <25 years and black/African American MSM could improve outcomes along the continuum of care.

Early awareness of HIV-positive status permits earlier entry into the continuum of care. In 2011, the National HIV Behavioral Surveillance System in 20 cities found that only 49% of MSM aged 18–24 years who tested positive were aware of their HIV infection (8). By race/ethnicity, 54% of black/African American, 63% of Hispanic/Latino, and 86% of white MSM aged ≥18 years who tested positive were aware of their infection. Persons who are aware of their HIV-positive status are less likely to engage in risky behaviors that increase the probability of transmitting HIV to sex partners (9) and can enter into care and treatment earlier, further improving health outcomes. CDC recommends routine HIV screening for all persons aged 13–64 years in health care settings. Health care providers should subsequently test all persons likely to be at high risk for HIV at least annually, including MSM (9). Among MSM who reported negative or unknown HIV status in 2011 in the National HIV Behavioral Surveillance System, 67% reported testing for HIV during the past year (10). Sexually active, HIV-negative MSM might benefit from more frequent testing, such as every 3–6 months (10).

The findings in this report are subject to at least four limitations. First, analyses based on the NHSS data are from 19 jurisdictions with complete CD4 and VL reporting. Data from these 19 areas might not be representative of data on all MSM diagnosed with HIV infection in the United States. Data from these areas represent 42% of MSM diagnosed with HIV infection. Second, overall national data might not be applicable to all states. Third, analyses in this study are based on different populations. Linkage to care and retention in care were based on data for persons aged ≥13 years from 19 jurisdictions, whereas ART prescription and viral suppression were based on weighted estimates of persons receiving care who were aged ≥18 years and resided in the United States and Puerto Rico. In addition, ART use should be interpreted in the context of U.S. Department of Health and Human Services guidelines¶¶ that were in effect at the time of data collection; ART might have been prescribed less frequently for those with CD4 counts >500 cells/mm^3^. Finally, documentation of the most recent viral load might not be indicative of consistent viral suppression in this population over time.

CDC has adopted a high-impact prevention approach to reduce the number of new HIV infections by using a combination of scientifically proven, cost-effective, and scalable interventions targeted to relevant populations and geographic areas for increasing the impact of HIV prevention efforts and achieving the goals of the National HIV/AIDS Strategy.*** CDC currently funds prevention, surveillance, research, and evaluation programs for a diverse range of MSM, including young racial/ethnic minority MSM.††† The findings in this report highlight the need for continued expansion of prevention, care, and treatment efforts for achieving improvement in linkage to care, retention in care, and viral suppression for MSM, particularly MSM aged <25 years and black/African American MSM. Given that MSM account for more than half of new infections and comprise approximately half of persons living with HIV infection, to reduce HIV incidence, improve health outcomes, and reduce HIV-related health disparities in the United States, the prevention and care needs of MSM must be addressed.

## Figures and Tables

**TABLE 1:** Linkage to HIV medical care within 3 months after HIV diagnosis during 2010,*† among men aged ≥13 years who have sex with men, by selected characteristics — National HIV Surveillance System, 19 jurisdictions,§ United States

Characteristic	No. of HIV diagnoses	Linkage to care¶	

		No.	(%)
**Age group at diagnosis (yrs)**			
13–24	2,764	1,962	(71.0)
25–34	3,128	2,383	(76.2)
35–44	2,227	1,822	(81.8)
45–54	1,491	1,254	(84.1)
≥55	484	405	(83.7)
**Race/Ethnicity**			
Black/African American	4,348	3,115	(71.6)
Hispanic/Latino**	2,060	1,653	(80.3)
White	3,165	2,624	(82.9)
Other††	521	434	(83.3)
**Total** §§	**10,093**	**7,826**	**(77.5)**

**Abbreviation:** HIV = human immunodeficiency virus.

* Data include persons with a diagnosis of HIV infection regardless of stage of disease at diagnosis.

† Data statistically adjusted to account for missing transmission categories.

§ The 19 jurisdictions were California (Los Angeles County and San Francisco only), Delaware, District of Columbia, Georgia, Hawaii, Illinois, Indiana, Iowa, Louisiana, Michigan, Minnesota, Missouri, Nebraska, New Hampshire, New York, North Dakota, South Carolina, West Virginia, and Wyoming.

¶ One or more CD4+ T-lymphocyte or viral load tests within 3 months after HIV diagnosis.

** Hispanic/Latino MSM can be of any race.

†† Includes American Indian/Alaska Native, Asian, Native Hawaiian/Other Pacific Islander, and multiple races.

§§ Estimates might not sum to total.

**TABLE 2:** Retention in HIV medical care among men aged ≥13 years who have sex with men and whose HIV infection was diagnosed by December 31, 2009,*† and who were alive on December 31, 2010, by selected characteristics — National HIV Surveillance System, 19 jurisdictions,§ United States

Characteristic	No. living with diagnosed HIV	Retention in care in 2010¶	

		No.	(%)
**Age group on December 31, 2009 (yrs)**			
13–24	7,775	3,552	(45.7)
25–34	26,793	12,788	(47.7)
35–44	52,086	26,331	(50.6)
45–54	59,128	30,765	(52.0)
≥55	28,288	15,132	(53.5)
**Race/Ethnicity**			
Black/African American	57,942	26,852	(46.3)
Hispanic/Latino**	34,254	18,515	(54.1)
White	74,150	38,630	(52.1)
Other††	7,663	4,559	(59.5)
**Total** §§ ¶¶	**174,071**	**88,569**	**(50.9)**

**Abbreviation:** HIV = human immunodeficiency virus.

* Data include persons with a diagnosis of HIV infection regardless of stage of disease at diagnosis.

† Data statistically adjusted to account for missing transmission categories.

§ The 19 jurisdictions were California (Los Angeles County and San Francisco only), Delaware, District of Columbia, Georgia, Hawaii, Illinois, Indiana, Iowa, Louisiana, Michigan, Minnesota, Missouri, Nebraska, New Hampshire, New York, North Dakota, South Carolina, West Virginia, and Wyoming.

¶ Two or more CD4+ T-lymphocyte or viral load tests performed at least 3 months apart during 2010.

** Hispanic/Latino MSM can be of any race.

†† Includes American Indian/Alaska Native, Asian, Native Hawaiian/Other Pacific Islander, and multiple races.

§§ Includes persons of unknown race/ethnicity.

¶¶ Estimates might not sum to total.

**TABLE 3:** ART prescription and viral suppression among men aged ≥18 years who have sex with men and whose HIV infection was diagnosed by December 31, 2009,*† and who were alive on December 31, 2010, by selected characteristics — National HIV Surveillance System, Medical Monitoring Project, United States and Puerto Rico

Characteristic	No. living with diagnosed HIV§	Antiretroviral therapy (ART) prescription¶		Viral suppression**	
	
		No.	(%)	No.	(%)
**Age group at interview (yrs)**					
18–24	18,792	5,740	(30.5)	4,872	(25.9)
25–34	63,931	27,006	(42.2)	20,608	(32.2)
35–44	125,164	51,920	(41.5)	44,881	(35.9)
45–54	143,034	77,258	(54.0)	64,792	(45.3)
≥55	65,808	44,537	(67.7)	40,039	(60.8)
**Race/Ethnicity**					
Black/African American	123,819	58,276	(47.1)	45,813	(37.0)
Hispanic/Latino††	82,410	40,509	(49.2)	34,233	(41.5)
White	195,086	96,787	(49.6)	85,657	(43.9)
Other§§	15,034	10,889	(72.4)	9,488	(63.1)
**Total** ¶¶ ***	**416,730**	**206,461**	**(49.5)**	**175,191**	**(42.0)**

**Abbreviations:** ART = antiretroviral therapy; HIV=human immunodeficiency virus.

* Data include persons with a diagnosis of HIV infection regardless of stage of disease at diagnosis.

† Data statistically adjusted to account for reporting delays and missing transmission categories.

§ National HIV Surveillance System estimates for United States and Puerto Rico.

¶ Medical Monitoring Project estimates for United States and Puerto Rico for persons who received medical care during January–April 2010 and who had documentation of ART prescription in the medical record.

** Medical Monitoring Project estimates for United States and Puerto Rico for persons who received medical care during January–April 2010 and whose most recent HIV viral load in the preceding 12 months was undetectable or ≤200 copies/mL.

†† Hispanic/Latino MSM can be of any race.

§§ Includes American Indian/Alaska Native, Asian, Native Hawaiian/Other Pacific Islander, and multiple races.

¶¶ Includes persons of unknown race/ethnicity.

*** Estimates might not sum to total.

## Footnotes

* NHSS is the primary source for monitoring HIV trends in the United States. The system collects, analyzes, and disseminates information about new and existing cases of HIV infection.

† MMP is a supplemental HIV surveillance system designed to produce nationally representative estimates of the prevalence of behavioral and clinical characteristics among HIV-infected adults aged ≥18 years receiving medical care in the United States and Puerto Rico.

§ The 19 jurisdictions were California (Los Angeles County and San Francisco only), Delaware, District of Columbia, Georgia, Hawaii, Illinois, Indiana, Iowa, Louisiana, Michigan, Minnesota, Missouri, Nebraska, New Hampshire, New York, North Dakota, South Carolina, West Virginia, and Wyoming. The criteria for complete reporting were as follows: 1) the jurisdiction’s laws or regulations required reporting of all CD4 and VL test results to the state or local health department, 2) ≥95% of all laboratory test results were reported by laboratories that conducted HIV-related testing for each jurisdiction, and 3) the jurisdiction reported to CDC all CD4 and VL results received since at least January 2010.

¶ Defined as having one or more CD4 (count or percentage) or VL test performed within 3 months after HIV diagnosis during 2010, including those performed during the same month as diagnosis.

** Defined as having two or more CD4 or VL results at least 3 months apart during 2010, among persons diagnosed through December 31, 2009, and alive on December 31, 2010.

†† ART prescription was based on MMP data for all MSM MMP participants in the 2010 data collection cycle.

§§ Viral suppression was based on all MSM MMP participants in the 2010 data collection cycle and was defined as having a VL result of ≤200 copies/mL at the most recent HIV VL in the preceding 12 months. The cut-off value of ≤200 copies/mL was based on the U.S. Department of Health and Human Services recommended definition of virologic failure.

¶¶ Additional information available at http://aidsinfo.nih.gov/contentfiles/lvguidelines/adultandadolescentgl.pdf.

*** Additional information available at http://www.cdc.gov/nchhstp/newsroom/hivfactsheets/future/high-impact-prevention.htm.

††† Additional information available at http://www.cdc.gov/msmhealth/msm-programs.htm.
